# Inflammation as a Precursor of Tumour Formation

**Published:** 1900-12-29

**Authors:** 


### INFLAMMATION AS A PRECURSOR OF TUMOUR FORMATION.

At the last meeting of the Pathological Society Mr. F. T. Paul drew attention to the tendency of chronic mastitis to end in tumour formation. It was known, he said, that cancerous breasts almost constantly showed microscopical changes indicating or preceding mastitis, and that breasts removed for simple chronic mastitis frequently presented appearances suggestive of the commencement of cancer; and, taking all the facts into consideration, the histological appearances as well as clinical experience, he said that it seemed certain that chronic inflammation of the breast, if not the actual cause, was at any rate the frequent predisposing cause of tumour change in this organ. Naturally the corollary of this position was that excision was desirable in all marked or inveterate cases of chronic mastitis. In the discussion which followed, however, Mr. Paul did not receive much support for his views.

